# PRedicting the EVolution of SubjectIvE Cognitive Decline to Alzheimer’s Disease With machine learning: the PREVIEW study protocol

**DOI:** 10.1186/s12883-023-03347-8

**Published:** 2023-08-12

**Authors:** Salvatore Mazzeo, Michael Lassi, Sonia Padiglioni, Alberto Arturo Vergani, Valentina Moschini, Maenia Scarpino, Giulia Giacomucci, Rachele Burali, Carmen Morinelli, Carlo Fabbiani, Giulia Galdo, Lorenzo Gaetano Amato, Silvia Bagnoli, Filippo Emiliani, Assunta Ingannato, Benedetta Nacmias, Sandro Sorbi, Antonello Grippo, Alberto Mazzoni, Valentina Bessi

**Affiliations:** 1 Department of Neuroscience, Psychology, Drug Research and Child Health, University of Florence, Azienda Ospedaliera-Universitaria Careggi, Largo Brambilla 3, Florence, 50134 Italy; 2 Research and Innovation Centre for Dementia-CRIDEM, Azienda Ospedaliero-Universitaria Careggi, Florence, Italy; 3 The BioRobotics Institute and Department of Excellence in Robotics and AI, Scuola Superiore Sant’Anna, Pisa, Italy; 4 Regional Referral Centre for Relational Criticalities - Tuscany Region, Florence, Italy; 5 IRCCS Fondazione Don Carlo Gnocchi, Florence, Italy

**Keywords:** Alzheimer’s disease, Subjective cognitive decline, Neuropsychology, Biomarkers, Electroencephalography, Event-related potential

## Abstract

**Background:**

As disease-modifying therapies (DMTs) for Alzheimer's disease (AD) are becoming a reality, there is an urgent need to select cost-effective tools that can accurately identify patients in the earliest stages of the disease. Subjective Cognitive Decline (SCD) is a condition in which individuals complain of cognitive decline with normal performances on neuropsychological evaluation. Many studies demonstrated a higher prevalence of Alzheimer’s pathology in patients diagnosed with SCD as compared to the general population. Consequently, SCD was suggested as an early symptomatic phase of AD. We will describe the study protocol of a prospective cohort study (PREVIEW) that aim to identify features derived from easily accessible, cost-effective and non-invasive assessment to accurately detect SCD patients who will progress to AD dementia.

**Methods:**

We will include patients who self-referred to our memory clinic and are diagnosed with SCD. Participants will undergo: clinical, neurologic and neuropsychological examination, estimation of cognitive reserve and depression, evaluation of personality traits, *APOE* and *BDNF* genotyping, electroencephalography and event-related potential recording, lumbar puncture for measurement of Aβ_42_, t-tau, and p-tau concentration and Aβ_42_/Aβ_40_ ratio. Recruited patients will have follow-up neuropsychological examinations every two years. Collected data will be used to train a machine learning algorithm to define the risk of being carriers of AD and progress to dementia in patients with SCD.

**Discussion:**

This is the first study to investigate the application of machine learning to predict AD in patients with SCD. Since all the features we will consider can be derived from non-invasive and easily accessible assessments, our expected results may provide evidence for defining cost-effective and globally scalable tools to estimate the risk of AD and address the needs of patients with memory complaints. In the era of DMTs, this will have crucial implications for the early identification of patients suitable for treatment in the initial stages of AD.

**Trial registration number (TRN):**

NCT05569083.

## Background

Research and clinical practice on Alzheimer's disease (AD) are at a turning point. The AD drug development pipeline is leading to new therapies [1], with aducanumab and lecanemab being the first disease-modifying therapies (DMTs) approved for AD [2, 3]. It is widely accepted that DMTs should be administered in the early stages of the disease to halt the pathological process before neurodegeneration begins [4]. Consequently, international research is now focusing on the prodromal and preclinical phases of AD. Subjective cognitive decline (SCD) refers to a self-experienced persistent decline in cognitive capacity compared to the previously normal state. During this decline, individuals exhibit normal age-, sex-, and education-adjusted performance on standardized cognitive tests [5].

SCD has been associated with neuroradiological features suggestive of AD, amyloid deposition [6, 7], and a higher risk of progression to Mild Cognitive Impairment (MCI) or dementia compared to individuals without SCD [8]. Recognizing these associations, the National Institute of Aging-Alzheimer's Association (NIA-AA) has included SCD as the first manifestation of symptomatic AD stages, preceding MCI [9]. Consequently, individuals with SCD may represent a target population for DMT to preserve cognitive function and psychological well-being [10].

However, SCD encompasses a heterogeneous group with various possible trajectories [11] and numerous potential underlying causes, including normal ageing, personality traits, psychiatric, neurological or medical disorders, substance use disorder, and medications [12]. Therefore, it is crucial to identify features and tools that accurately detect prodromal AD among patients with SCD.

In recent years, the Regional Reference Centre for Alzheimer's Disease and Cognitive Disorders of Careggi Hospital in Florence, Italy, has analysed a large dataset of neuropsychological, personality, and lifestyle data collected over approximately 25 years from patients with SCD. This analysis identified demographic [13, 14], cognitive [15, 16], personality [15], and genetic [15] and genetic [13, 17–23] features that increase the risk of progression from SCD to MCI or AD.

This paper describes the protocol of the PREVIEW (PRedicting the EVolution of SubjectIvE Cognitive Decline to Alzheimer's Disease With machine learning) study, which will prospectively investigate baseline predictors and biomarkers of Alzheimer's pathology and progression to MCI and dementia in a large cohort of patients with SCD. In this study, we will integrate our previous findings with data from non-invasive techniques, such as electroencephalography (EEG) and event-related potentials (ERP) recording. These techniques reliably measure neural circuits associated with cognitive processes and may provide sensitive metrics for early diagnosis of cognitive impairment [24]. Additionally, we will employ machine learning approaches, an emerging and promising tool that has demonstrated great potential in diagnosing and classifying neurodegenerative diseases and other medical conditions [25–27].

Specifically, our aims are as follows:

i) Integrate a multimodal set of data from SCD patients, including clinical data, neuropsychological assessments, personality traits, cognitive reserve, genetic factors, and features from EEG and ERP recordings.
ii) Train and test a machine learning model based on these features to predict biological AD pathology (defined according to CSF biomarkers) and conversion from SCD to MCI and AD dementia through machine learning tools.
iii) Define a management protocol for SCD to be applied in memory clinic settings.

The PREVIEW study was registered on ClinicalTrials.gov (registration number: NCT05569083).

## Methods and analysis

### Study design and participants

This is a longitudinal observational cohort study. We will include consecutive patients who self-referred to the Centre for AD and Adult Cognitive Disorders of Careggi Hospital in Florence and are classified as SCD based on SCD-I criteria [5].

We will recruit patients who meet the following criteria:

I age between 45 and 90 years;
II complaining of cognitive decline with a duration of ≥ 6 months;
III Mini Mental State Examination (MMSE) score greater than 24, corrected for age and education;
IV normal functioning on the Activities of Daily Living (ADL) and the Instrumental Activities of Daily Living (IADL) scales [28];
V unsatisfied criteria for MCI [29] and dementia [30];

Exclusion criteria are history of head injury, current neurological and/or systemic disease, symptoms of psychosis, major depression, or substance use disorder (as defined by the Diagnostic and Statistical Manual of Mental Disorders, Fifth Edition [DSM-5] [30]).

The evaluation of exclusion criteria will be conducted through a thorough neurological examination of the patients. The presence of exclusion criteria will be assessed by two independent neurologists. In the event of a disagreement, a third neurologist will be consulted to reach a consensus.

All recruited patients will undergo the following assessments at baseline (T_0_):

I comprehensive evaluation of familial and clinical history;
II extensive neuropsychological assessment, including estimation of premorbid intelligence, evaluation of depression, personality assessment, and assessment of leisure activities;
III blood collection for measurement of vitamin B12, folic acid, thyroid hormones, as well as *APOE* and *BDNF* genotype analysis;
IV EEG and ERP recording.

Patients who provide additional informed consent for lumbar puncture will undergo CSF collection to measure Aβ_42_, Aβ_42_/Aβ_40_ ratio, total tau (t-tau), and phosphorylated tau (p-tau).

Patients will undergo neuropsychological evaluations every two years until progression to AD or other dementias. Based on the results of previous meta-analysis [8], we estimate that a period of six years will be required to reach the desired sample size (see Sect. 2.2). Progression to MCI and AD dementia will be determined based on the criteria established by the NIA-AA [29, 31]. Patients who progress to dementia will be referred to our centre for diagnostic and therapeutic assessments.

**Fig. 1:**
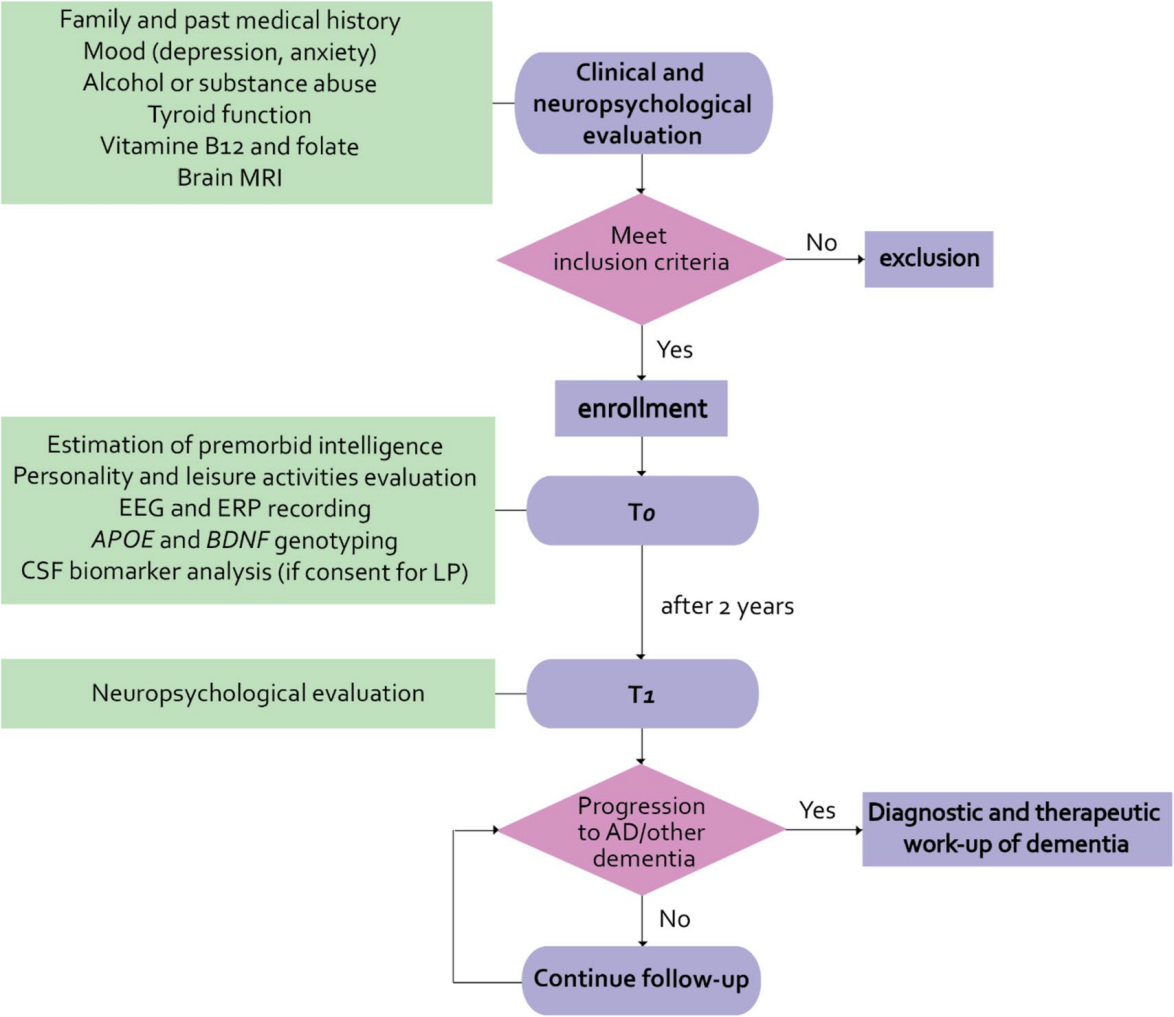
Flowchart summarizing the design of the study. Abbreviations: CSF = cerebrospinal fluid; EEG = electroencephalogram; ERP = event-related potentials; AD = Alzheimer’s disease; *BDNF* = brain-derived neurotrophic factor; *APOE* = apolipoprotein

A summary of the study design is shown in Fig. 1.

For the purpose of cross-sectional comparison with the SCD group at baseline, two samples consisting of age-matched healthy controls (without cognitive concerns) and MCI patients will undergo EEG and ERP recording.

### Sample size calculation

We estimated the sample size needed for this study using the statistical power of a hypothesis test using the Python module statsmodels [32]. We considered the following parameters:

- Level of significance (α) = 0.05
- Statistical power (1-β) = 0.8
- Effect size (Cohen's d) = 0.6

The required number of patients was 45 per group. Therefore, considering that we aim to compare patients who will show a progression to objective cognitive decline and patients who will not, the total estimated sample size should be 90 individuals.

With an expected conservative drop-out rate of 10%, the number of participants to be included was estimated to be 99 patients.

### Neuropsychological evaluation, assessment of depression and estimation of premorbid intelligence

Extensive neuropsychological examination includes: global measurements (MMSE) [33], tasks exploring verbal and spatial short-term and long-term memory (Digit and Visuo-spatial Span forward and backward [34], Rey Auditory Verbal Learning Test [35], Short Story Immediate and Delayed Recall [36], Rey-Osterrieth complex figure recall [37]), attention (Trail Making Test A [38], attentional matrices [39], Multiple Features Targets Cancellation [40]), language (Category Fluency Task [41], Phonemic Fluency Task [35] and Italian language battery: Screening for Aphasia NeuroDegeneration [42]), constructional praxis (Copying drawings [35], Rey-Osterrieth complex figure copy [37], Clock test [43]) and executive function (Trail Making Test B [38], Stroop Test [44], Frontal Assessment Battery [45]). The subjective perception of memory impairment will be investigated using the Memory Assessment Clinics-Questionnaire (MAC-Q) [46]. Premorbid intelligence will be estimated using the Short intelligence test (TIB) [47], that has been constructed as the Italian equivalent of the National Adult Reading Test [48]. The presence and severity of depressive symptoms will be evaluated by means of the 22-item Hamilton Depression Rating Scale (HDRS) [49]. Katz Index of Independence in Activities of Daily Living (ADL) [50] scale will be used to assess functional capacities at baseline and at follow-up (Table 1).

**Table 1:** Explored cognitive domains and respective neuropsychological tests

Cognitive domains	Neuropsychological tests	References
Global cognition	MiniMental-State Examination (MMSE)	(Magni et al. 1996) [33]
Verbal short-term memory	Digit span forward and backward	(Monaco et al. 2013) [34]
Verbal long-term memory	Rey Auditory Verbal Learning Test (RAVLT)	(Carlesimo, Caltagirone, e Gainotti 1996) [35]
	Short story recall	(De Renzi, Faglioni, e Ruggerini 1977) [36]
Spatial short-term memory	Visuo-spatial span forward and backward	(Monaco et al. 2013) [34]
Spatial long-term memory	Rey-Osterrieth complex figure recall	(P. Caffarra et al. 2002) [37]
Attention	Attentional matrices	(Della Sala et al. 1992) [39]
	Trail Making Test part A	(Giovagnoli et al. 1996) [38]
	Multiple Features Targets Cancellation	(Marra et al. 2013) [40]
Language	Category fluency task	(Novelli et al. 1970) [41]
	Phonemic fluency task	(Carlesimo, Caltagirone, e Gainotti 1996) [35]
	Screening for Aphasia in NeuroDegeneration (SAND)	(Catricalà et al. 2017) [42]
Constructional praxis	Copying drawings	(Carlesimo, Caltagirone, e Gainotti 1996) [35]
	Rey-Osterrieth complex figure copy	(P. Caffarra et al. 2002) [37]
	Clock test	(Shulman et al. 1993) [43]
Executive function	Trail Making Test part B	(Giovagnoli et al. 1996) [38]
	Stroop Test	(Paolo Caffarra et al. 2002) [37, 44]
	Frontal Assessment Battery	(Appollonio et al. 2005) [45]
Perception of memory impairment	Memory Assessment Clinics-Questionnaire (MAC-Q)	(Crook, Feher, e Larrabee 1992) [46]
Premorbid intelligence	Short intelligence test (TIB)	(Colombo et al. 2000) [47]
Depression	Hamilton Depression Rating Scale	(Hamilton 1960) [49]
Functional capacities	Katz Index of Independence in Activities of Daily Living	(Katz et al. 1963) [50]

### Personality traits and leisure activities

We will use the Big Five Factors Questionnaire (BFFQ) [51] to assess personality traits. Participants will be asked to fill out a questionnaire that measures the five factors of: 1) emotional stability, 2) energy, 3) conscientiousness, 4) agreeableness, and 5) openness to culture and experience. The inventory follows a widely accepted five-traits personality model [51, 52]. For the 24 items of each factor, subjects will rate their level of agreement on a five-point scale ranging from strongly agree to strongly disagree. Item scores will be computed for each factor to yield a summary measure of the trait with higher values representing a greater degree of the explored dimension.

At baseline, subjects will be interviewed regarding participation, when they were 30–40 years old, in nine Intellectual Activities, seven Social Activities and seven Physical Activities (modified from Yarnold PR et al. [53]). The frequency of participation will be reported for each activity on a Likert scale ranging from 0 to 5, where 0 refers to never, 1 to occasionally, 2 to monthly, 3 to once a week, 4 to several days per week and 5 to daily.

### EEG and ERP recording

Resting-state EEG data will be collected at the Neurophysiological Laboratory of IRCCS Don Gnocchi (Florence, Italy) using the 64-channel Galileo-NT system (E.B. Neuro S.p.A.). The EEG will be recorded continuously from 64 electrodes using an EEG Prewired Headcaps. Electrodes were positioned according to the 10–10 international system (AF7, AF3, Fp1, Fp2, Af4, Af8, F7, F5, F3, F1, F2, F4, F6, F8, FT7, FC5, FC3, FC1,FC2, FC4, FC6, FT8, T3, C5, C3, C1, C2, C4, C6, T4 TP7, CP5, CP3, CP1, CP2, CP4, CP6, TP8, T5, P5, P3, P1, P2, P4, P6, T6, Fpz, PO7, PO3, O1, O2, PO4, PO8, Oz, AFz, Fz, FCz, Cz, CPz, Pz, and POz). The ground electrode will be placed in front of Fz. Horizontal eye movements will be detected by electrooculogram (EOG). Data will be digitized at a sampling rate of 512 Hz and analogue–digital precision will be 16 bits. The recording will be referenced to the common average of all electrodes, excluding Fp1 and Fp2. Re-referencing will be done prior to the EEG artifact detection and analysis. Electrode–skin impedance will be set below 5 kΩs. Subjects will be seated in a reclined chair in a comfortable position. Resting EEG recording begins with a 10-min eyes-closed registration followed by an alternance of 3 min eyes-open and 3 min eyes closed, repeated twice. Only the eyes-closed portions of the signal will be used for subsequent analyses.

ERP acquisition will be performed with the same EEG system used for EEG data acquisition. The participants will be administered an ERP test battery with concurrently recorded EEG consisting of a 3-choice vigilance task (3CVT) and standard image recognition task (SIR).

In order to remove electrophysiological and non-electrophysiological artifacts from the raw signals, we will apply a custom preprocessing pipeline written in MATLAB with the use of the EEGLAB toolbox functions [54]. The pipeline consists of two main steps: the PREP pipeline [55], followed by independent component analysis (ICA) to remove artefactual components [56]. Moreover, to generate the ERP epochs, time windows from -300 ms to + 1000 ms will be created for each EEG recording channel, with the stimulus presentation centred at 0 ms and the average of the trials from each epoch will be calculated.

### Blood sample collection and analysis of *APOE* and *BDNF* genes

Blood samples will be collected by venipuncture into standard polypropylene EDTA test tubes (Sarstedt, Nümbrecht, Germany) at the Neurology Unit of Careggi University Hospital. They will be centrifuged within two hours at 1300 rcf at room temperature for 10 min, and plasma will be isolated and stored at -80 °C until tested at the Laboratory of Neurogenetics at Careggi University Hospital.

*APOE* genotypes will be investigated by HRMA. The samples with known *APOE* genotypes, which had been validated by DNA sequencing, will be used as standard references.

Analysis of *BDNF* rs6265 polymorphism will be performed using HRMA and the genotypes will be identified through Sanger sequencing (SeqStudio Genetic Analyzer, ThermoFisher).

### CSF collection and AD biomarker measurement

CSF samples will be collected at 8:00 a.m. by lumbar puncture at the Neurology Unit of Careggi University Hospital. Samples will be immediately centrifuged and stored at -80 °C until performing the analysis at the Laboratory of Neurogenetics of Careggi University Hospital. Aβ_42_, Aβ_40_, t-tau, and p-tau will be measured using a chemiluminescent enzyme immunoassay (CLEIA) analyser LUMIPULSE G600 (Fujirebio, Tokyo, Japan). Cut-off values for CSF biomarkers will be determined following Fujirebio guidelines (diagnostic sensitivity and specificity using clinical diagnosis and the follow-up golden standard as of November 19^th^, 2018).

### Data collection and management

Data collection will be carried out anonymously on REDCap, an online-based software for the design of databases. Data will be collected in a pseudo-anonymized way, attributing a record ID to each patient on the electronic database and saving the correspondence between names and identification codes on a separate document.

### EEG pre-processing

The EEG preprocessing will be conducted following the methodology outlined in Lassi et al., 2023 [57]. Firstly, the PREP pipeline will be applied, which involves high-pass filtering the signals at 1 Hz, removing line noise using the CleanLine routine, and identifying noisy channels through a combination of methods. Following the interpolation of noisy channels, the data will be re-referenced to the median. Subsequently, Independent Component Analysis (ICA) [58] will be performed on the re-referenced signals to eliminate artifacts while preserving neural components. To classify the components as neural or artefactual, a semi-automated process will be employed, utilizing both the ICLabel [59] toolbox and visual inspection. After completing these two processing steps, a final visual inspection will be conducted to identify and remove any remaining artifacts, if present.

### EEG Statistical analysis

Firstly, we will calculate the power spectral density (PSD) of the signal recorded in each channel using the Welch's method. The PSD will be computed on continuous windows of EEG signals, applying Hanning windows with no overlap. The spectrum will be divided into four canonical frequency bands: delta (1–4 Hz), theta (4–8 Hz), alpha (8–13 Hz), and beta (13–30 Hz). The scalp will be divided into six regions of interest (ROIs): frontal right (Fp2, AF4, AF8, F2, F4, F6, F8), frontal left (Fp1, AF3, AF7, F1, F3, F5, F7), central right (FC2, FC4, FC6, FT8, C2, C4, C6, T4, CP2, CP4, CP6), central left (FC1, FC3, FC5, FT7, C1, C3, C5, T3, CP1, CP3, CP5), occipital right (P2, P4, P6, T6, PO8, PO4, O2), and occipital left (P1, P3, P5, T5, PO7, PO3, O1). ROI power will be computed as the average relative power from channels belonging to each ROI.

Next, we will analyse the connectivity between pairs of ROIs using LORETA source-reconstructed signals and extract several network metrics from the weighted undirected adjacency matrices. These metrics will include the average strength of connectivity among pairs of ROIs (mean weight of non-zero connections), the weighted clustering coefficient (C) and the weighted characteristic path length (L) [60]. Finally, we will compute the small-world coefficient [57]:

$$\Omega = \frac{Lr}{L}- \frac{C}{Cl}$$

where L and C are the previously computed weighted clustering coefficient and weighted characteristic path length, whereas Lr is the weighted characteristic path length of an equivalent random network and Cl is the weighted clustering coefficient of an equivalent lattice network.

The final statistical analysis will involve extracting microstate maps [61] for each subject individually. Initially, a set of common microstates will be extracted from all subjects, regardless of conditions, using the modified k-means algorithm [62]. Then, individual microstate maps will be generated, and the template maps will be matched with the previously obtained grand-averages. The EEG signals of each subject will be reconstructed as a sequence of microstates by assigning each topography to the most similar microstate average map. From the reconstructed microstate sequence, various metrics such as duration, transition probability, and complexity of the sequence will be used as features. Spectral features, network metrics, and microstate features will be compared across conditions, providing the foundation for the machine learning analysis.

Regarding the ERP data, both spectral features (like the EEG processing) and voltage-level signals (amplitude, latency, integral of the signal) from different phases of the ERP will be used as input features.

### Descriptive statistical analysis

Descriptive statistical analyses will be conducted using IBM SPSS Statistics Software Version 25 (SPSS Inc., Chicago, USA) and the computing environment R4.2.3 (R Foundation for Statistical Computing, Vienna, 2013). The distributions of variables will be assessed using the Shapiro–Wilk test. Patient groups will be characterized using means and standard deviations, medians and interquartile ranges (IQR), frequencies or percentages, and 95% confidence intervals (95% C.I.) for variables with continuous distribution, continuous non-normally distributed variables, and categorical variables, respectively. Depending on the distribution of the data, we will employ ANOVA or non-parametric Kruskal–Wallis tests for between-group comparisons, and Pearson's or Spearman's correlation coefficient to assess correlations between numeric measures of the groups. Chi-square tests will be used to compare categorical data. Effect sizes will be computed using Cohen's *d* for normally distributed numeric measures, η^2^ for the Mann–Whitney-U Test, and Cramer's *V* for categorical data.

### Machine learning classification of patient conditions

Demographic, clinical, cognitive, neurophysiological, and genetic data will be used as candidate input features without any further processing for training a machine learning model. During the training process, a feature selection algorithm will be applied to select the most informative features based on their performance on the validation set.

The machine learning problem will initially be treated as a multi-class classification task. The model will classify patients who are SCD at the time of the measurements into one of the following three classes: stable SCD, progressed to MCI, or progressed to AD dementia. For the subgroup of patients undergoing CSF biomarker analysis, a classification will be performed based on the results of the analysis. Specifically, patients will be classified as carriers or non-carriers of AD pathology, according to the NIA-AA framework [9].

For feature selection, we will test the efficacy of several algorithms including the ANOVA F-test statistic, ReliefF, mutual information, and minimum redundancy maximum relevance (MRMR). The number of retained features for each algorithm will be optimized in the validation loop.

Regarding the classifier models, a set of different machine learning models will be tested, including support vector machine (SVM) with linear, quadratic, cubic, and radial basis function kernels; random forest (RF); gradient boosted trees (xgboost); linear and quadratic discriminant analysis (LDA and QDA); and an artificial neural network (ANN). The hyperparameters of each algorithm will be optimized to determine the model with the best performance in the classification task.

In the SVM, the parameters C (misclassification penalty) and gamma (influence of single training points) will be optimized. For the RF, the number of trees (estimators), maximum depth of each tree, and minimum number of data points allowed in a leaf node will be optimized. The same hyperparameters will be tuned for xgboost, along with the learning rate of the algorithm. In LDA and QDA, the type of solver used by the algorithm and the shrinkage term will be adjusted. For the ANN, the number of layers and neurons per layer will be optimized. We have chosen to compare feature selection methods from classical statistics (such as ANOVA F-test) with data-driven methods (ReliefF and MI) that do not assume the distribution of the data. Additionally, the MRMR algorithm will be tested to consider possible redundancies among features.

In the machine learning models, both linear (linear SVM and LDA) and non-linear (polynomial and radial basis function [RBF]-SVM and QDA) algorithms that define separating hyperplanes will be tested, along with tree-based methods like RF and xgboost. The ANN will serve as a non-linear, black-box model to assess the discrimination performance and potential overfitting of more complex models. Furthermore, standard dimensionality reduction techniques will be applied to extract the most salient features. The procedure described above will be repeated to evaluate whether similar results can be achieved with a subset of the features. Once the cross-sectional classification is performed, longitudinal data will be utilized to develop an algorithm capable of predicting the future progression of cognitive impairment.

Out of the entire dataset, 30% of the data will be reserved as a testing set, while the remaining 70% will be used for training and validation of the model. A fivefold cross-validation approach will be employed to train and optimize the hyperparameters of the models. The best performing model, determined by the prediction F1-score and the set of optimized hyperparameters, will be tested on the testing set to obtain an unbiased estimate of the model's performance.

**Fig. 2:**
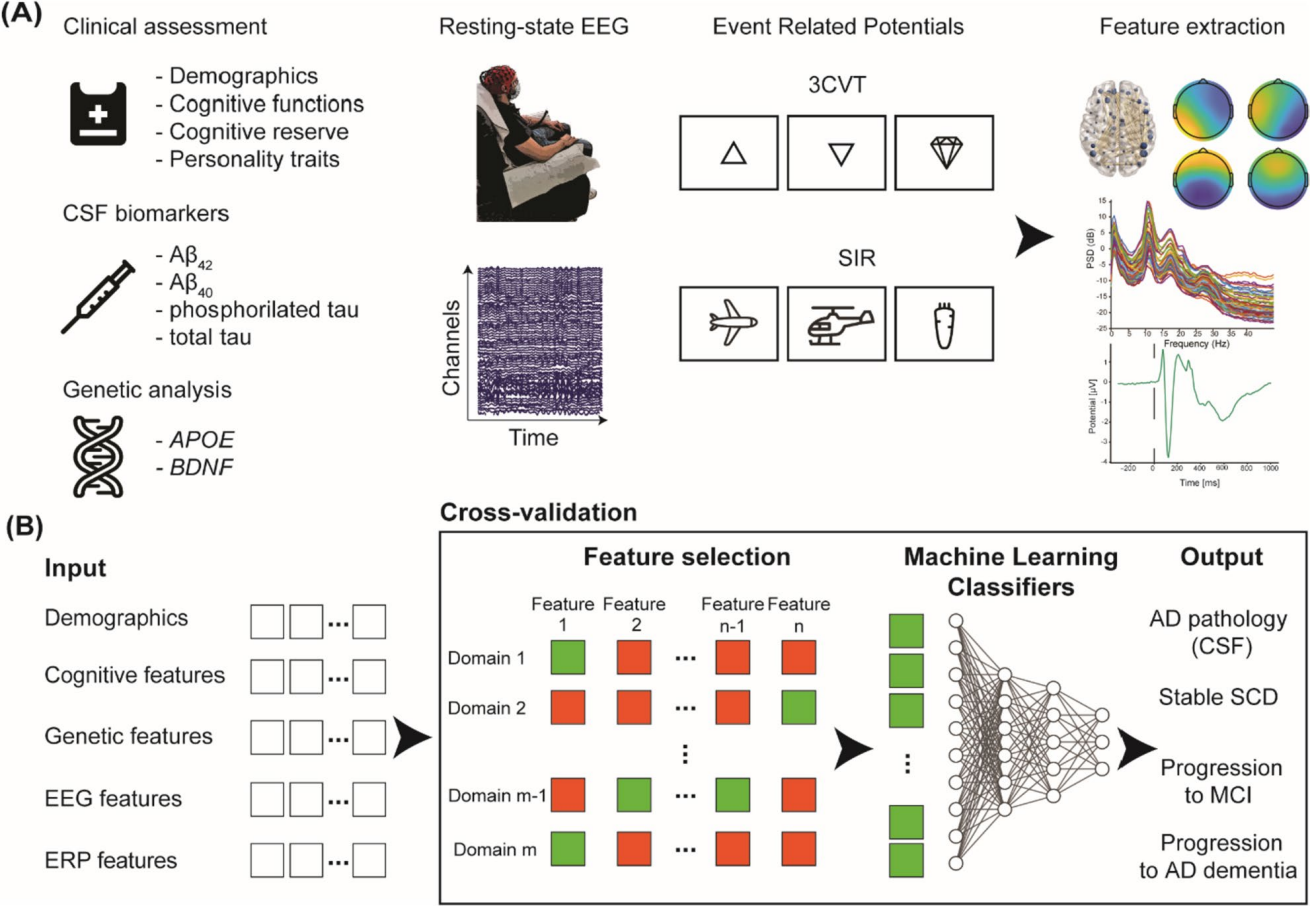
Visual summary of data collection and analysis. **A** Data collection and feature extraction. Multi-modal data is collected from the recruited patients: clinical-neuropsychological evaluations, genetic and biological data, EEG at rest and during memory and attention tasks (ERP). From the EEG signals, features are extracted by using several analyses, such as connectivity (top left), microstates (top right), spectral and ERP time course analyses. **B** The extracted features, candidate biomarkers of progression to AD, are submitted to the cross-validated machine learning framework. First, only informative features are selected (green squares), while the non-informative ones are discarded. The selected features are used as inputs to train a set of machine learning classifiers (e.g., an ANN is displayed) to determine whether: i) the subject is carrier of biological AD pathology; ii) will remain SCD or will progress towards MCI or AD dementia. Abbreviations: CSF = cerebrospinal fluid; EEG = electroencephalogram; ERP = event related potentials; 3 CVT = 3-choice vigilance task; SIR = standard image recognition task; SCD = subjective cognitive decline; MCI = mild cognitive impairment; AD = Alzheimer’s disease; *BDNF* = brain-derived neurotrophic factor; *APOE* = apolipoprotein E

A visual summary of the data collection and analysis process is shown in Fig. 2.

## Discussion

On June 7, 2021, the FDA provisionally approved aducanumab, the first anti-amyloid monoclonal antibody for treating patients with MCI due to AD and mild AD dementia [63]. This approval marks a significant milestone as it is the first disease-modifying therapy for AD. Furthermore, additional AD treatments may become available in the near future [1]. As a consequence, clinicians, researchers, and health services will face increasing demands for diagnostic assessments of patients with cognitive disorders. However, these treatments are not without risks, and the most common adverse effect is amyloid-related imaging abnormality (ARIA) [64].

In this context, there is an urgent need to select cost-effective and easily accessible tools for identifying patients in the early stages of the disease, while minimizing the inclusion of patients who will not progress to AD dementia. The currently recognized AD biomarkers, such as PET neuroimaging [65–67] or CSF biomarkers [68, 69], are expensive, invasive, and not suitable for large-scale application. Our study aims to address these limitations by considering features that can be collected through clinical and neuropsychological examinations, as well as non-invasive assessments like EEG and blood collection.

Moreover, it is crucial to consider the target population for screening purposes. Conducting a general population screening may lead to an unacceptable number of false positive results and subsequent costs. Therefore, we focus our attention on patients SCD, who are individuals referred to memory clinics. There is increasing evidence that patients with SCD have a higher risk of carrying Alzheimer's pathology and progressing to dementia [6–8], compared to individuals without SCD [8]. Thus, patients with SCD represent an optimal population for screening prodromal AD.

The PREVIEW study will collect demographic, personality, cognitive, genetic, EEG, and ERP data to train a machine learning algorithm. Previous studies have successfully employed this approach to predict dementia in non-demented populations [70, 71], as well as positive AD biomarkers in patients with MCI [72, 73], indicating the great potential of this approach. However, only a few studies have focused on predicting the progression of cognitive decline [74–77], and to our knowledge, no studies have applied machine learning to predict progression from SCD to dementia.

This approach offers several advantages over classical statistics. Firstly, it allows us to consider all the collected variables simultaneously. Secondly, the machine learning procedure helps identify the most informative features for screening, enabling the development of a protocol that reduces the burden on patients and healthcare costs associated with cognitive assessments.

Furthermore, this approach will provide additional evidence to clarify the role of certain variables that still have controversial associations with SCD. For example, numerous studies have shown that individuals with SCD have lower scores on neuropsychological tests compared to those without SCD [78–80]. However, only a few longitudinal studies have assessed the prognostic value of baseline neuropsychological assessments, often yielding conflicting results [81–84].

Several studies have focused on the relationship between SCD and cognitive reserve [85–89]. Engaging in intellectual activities during earlier decades and having higher premorbid intelligence have been identified as protective factors, reducing the risk of progression from SCD to MCI [15, 16]. However, cognitive reserve seems to have a dual effect, as individuals with higher cognitive reserve exhibit faster disease progression once it begins [16, 90]. The interaction between SCD and mood disorders is also controversial. Recent community-based studies on large populations have shown that depressive symptoms increase the risk of progression to objective cognitive decline and dementia in SCD patients [91, 92]. However, a meta-analysis by Huang et al. found that depression was significantly higher in individuals with SCD compared to normal individuals, but there was no difference between SCD and MCI, or between SCD converters and non-converters [93]. Studies on personality traits have yielded conflicting results regarding their association with SCD. Most studies agree that high conscientiousness and low neuroticism are associated with a reduced risk of incident AD [94, 95]. However, a previous study found that emotional stability was significantly higher in SCD patients who progressed to MCI or AD dementia [96].

In the past decades, EEG has been extensively evaluated as a diagnostic tool for dementia [97–99]. However, previous studies have mostly focused on identifying quantitative EEG markers of AD compared to healthy controls. These markers can be classified into four main categories: i) spectral markers, ii) connectivity and network metrics, iii) complexity measures, and iv) microstates [100]. Regarding spectral markers, AD and MCI groups have shown slowing in oscillations of EEG activity, characterized by a decrease in higher frequency activity or an increase in low-frequency power, compared to healthy controls [101–103]. Other studies have demonstrated a reduction in the complexity of the EEG signal throughout the development of dementia [104–106]. Connectivity studies have investigated covariation patterns in EEG sensor or source signals, revealing a decrease in connectivity between brain areas, particularly in higher frequency bands, as cognitive impairment progresses [107–110]. More recently, event-related potentials (ERPs) have been suggested as potential sensitive and robust biomarkers for tracking disease progression and evaluating response to therapy [111, 112]. To the best of our knowledge, only a few studies have described quantitative EEG changes in patients with SCD [113–115], and ERP has not been investigated in this population thus far.

Our project has some limitations: i) CSF will not be available for all the patients; ii) neuroimaging techniques will be used only for basal assessment of patients and will not be considered for machine learning analysis; iii) healthy controls will undergo only EEG and ERP recording.

The PREVIEW study also has several strengths that deserve emphasis. As mentioned earlier, the use of machine learning is a significant strength and the primary innovative outcome of our project. Another strength is the collection of AD CSF biomarkers, which serves two main purposes: i) given that previous studies have reported an average ten-year period for SCD patients to develop dementia [8, 96], a long follow-up will be necessary to obtain an adequate sample size of patients who progress to AD. By using CSF biomarkers, we can identify patients at a higher risk of AD dementia (according to the ATN system [9]) and classify them as prodromal AD. This classification can be considered as a surrogate target for preliminary cross-sectional analyses; ii) as mentioned earlier, SCD can also be the first clinical manifestation of medical conditions other than AD. CSF biomarkers will enable us to differentiate SCD with Alzheimer's pathology from SCD due to other causes. It should be noted that our study includes patients who self-refer to our memory clinic, as recommended by previous studies, to reduce sample heterogeneity and increase the chances of identifying subjects with preclinical AD compared to community-based studies [116]. Finally, we would like to emphasize that all the features considered as potential predictors will be derived from non-invasive, relatively inexpensive, and easily accessible techniques.

## Conclusions

The advent of DMTs will bring about a significant shift in the management of patients with cognitive decline caused by AD. Since the efficacy of these drugs is closely tied to the disease stage, it is crucial for clinicians to be able to identify individuals at risk of AD before neurodegeneration sets in. A suitable target population for this purpose is individuals who present with cognitive complaints but lack objective evidence of impairment. The PREVIEW study aims to extensively characterize patients with SCD through clinical, neuropsychological, neurophysiological, and genetic assessments. Utilizing a machine learning approach, we aim to assess potential biomarkers and develop a robust predictive model to evaluate the risk of progression to AD. This approach will provide relevant evidence regarding the most significant features to be assessed as the initial step in the diagnostic pathway for patients with SCD, before confirming the presence of AD pathology through more invasive and expensive tests.

## Data Availability

The datasets used and/or analysed during the current study are available from the corresponding author on reasonable request.

## Abbreviations

3CVT: 3-Choise vigilance task
AD: Alzheimer's disease
ADL: Activities of Daily Living
ANN: Artificial neural network
*APOE*: Apolipoprotein E
*BDNF*: Brain-derived neurotrophic factor
BFFQ: Big Five Factors Questionnaire
CLEIA: Chemiluminescent enzyme immunoassay
CSF: Cerebrospinal fluid
DMTs: Disease-modifying therapies
EEG: Electroencephalography
ERP: Event-related potentials
HDRS: Hamilton Depression Rating Scale
HRMA: High-resolution melting analysis
IADL: Instrumental Activities of Daily Living
IQR: Interquartile range
LDA: Linear discriminant analysis
MAC-Q: Memory Assessment Clinics-Questionnaire
MCI: Mild Cognitive Impairment
MMSE: Minimental state examination
NIA-AA: National Institute of Aging-Alzheimer's Association
p-tau: Phosphorylated tau
PREVIEW: PRedicting the EVolution of SubjectIvE Cognitive Decline to Alzheimer's Disease With machine learning
PSD: Power spectral density
QDA: Quadratic discriminant analysis
REDCap: Research Electronic Data Capture
RF: Random forest
ROIs: Regions of interest
RBF: Radial basis function
SCD: Subjective cognitive decline
SIR: Standard image recognition
SVM: Support vector machine
TIB: Test di intelligenza breve
t-tau: Total tau
